# Hypertension care cascade in Chile: a serial cross-sectional study of national health surveys 2003-2010-2017

**DOI:** 10.1186/s12889-020-09483-x

**Published:** 2020-09-14

**Authors:** Álvaro Passi-Solar, Paula Margozzini, Jennifer S. Mindell, Milagros Ruiz, Carlos A. Valencia-Hernandez, Shaun Scholes

**Affiliations:** 1 Research Department of Epidemiology and Public Health, University College London, 1-19 Torrington Place, London, WC1E 6BT UK; 2 Department of Public Health, School of Medicine, Pontificia Universidad Católica de Chile, Diagonal Paraguay 362, CP 88330077 Santiago, Chile

**Keywords:** Chile, Hypertension, Blood pressure, Care cascade, Management, Awareness, Antihypertensive treatment, Health surveys, Guidelines

## Abstract

**Background:**

Trend data on hypertension prevalence and attainment indicators at each step of the care cascade (awareness, treatment, control) are required in Chile. This study aims to quantify trends (2003–2017) in prevalence and in the proportion of individuals with hypertension attaining each step of the care cascade among adults aged 17 years or older, and to assess the impact of lowering the blood pressure (BP) thresholds used to define elevated BP on these indicators.

**Methods:**

We used data from 2003, 2010, and 2017 Chilean national health surveys. Each year we assessed levels of (1) mean systolic (SBP) and diastolic (DBP) blood pressure, (2) hypertension prevalence (BP ≥ 140/90 mmHg or use of antihypertensive treatment), and (3) awareness, treatment, and control. Logistic regression on pooled data was used to assess trends in binary outcomes; linear regression was used to assess trends in continuous SBP and DBP. We compared levels of hypertension prevalence using two sources to ascertain antihypertensive treatment (self-reported versus medicine inventory). The 2017 American College of Cardiology/American Heart Association (ACC/AHA) guidelines were used to re-define hypertension using lower thresholds (BP ≥ 130/80 mmHg).

**Results:**

Hypertension prevalence was 34.0, 32.0 and 30.8% in 2003, 2010 and 2017, respectively. Levels of treated- and controlled-hypertension were significantly higher in 2017 than in 2003 (65% versus 41% for treatment, *P* < 0.001; 34% versus 14% for control, *P* < 0.001), while levels of awareness were stable (66% versus 59%, *P* = 0.130). Awareness, treatment, and control levels were higher among females in 2003, 2010, and 2017 (*P* < 0.001). Mean SBP and DBP decreased over the 15-year period, except for SBP among females on treatment. Adopting the 2017 ACC/AHA guidelines would increase hypertension prevalence by 17 and 55% in absolute and relative terms, respectively.

**Conclusions:**

Chile has experienced a positive population-wide lowering in blood pressure distribution which may be explained partly by a significant rise in levels of treated- and controlled-hypertension since 2003. Lowering the thresholds used to define elevated BP would substantially increase the financial public health challenge of further improving attainment levels at each step of the care cascade. Innovative and collaborative strategies are needed to improve hypertension management, especially among males.

## Background

Hypertension continues to be one of the most important health challenges, being a major risk factor for cardiovascular morbidity and mortality worldwide. Low and middle-income countries (LMICs) are showing the sharpest increase in the prevalence of hypertension globally during the last four decades [1], explained only partially by population ageing. LMICs also show lower levels of attainment at each step of the hypertension care cascade, compared with high-income countries (HICs) [2]. In Chile, cardiovascular diseases (CVD) are the main cause of death, explaining 26–27% of the overall mortality every year between 2000 and 2017 [3]. Chile, a country of 18.7 million inhabitants with 11.4% aged 64 years and over and with 37.4% living in the Metropolitan region (Santiago), has currently one of the most prosperous economies in Latin America (ranked as a HIC since 2012) but at the same time has a very low level of health expenditure (8.9% of GDP) [4]. Furthermore, this economic prosperity has not been shared equally across all segments of the population, creating a unique profile for hypertension prevalence and its care cascade (i.e. the proportion of persons with hypertension at various stages of the management continuum that starts with screening and ends with control).

 Chile has a very strong and efficient high coverage public-health care system bringing success in indicators of maternal and infant health. Sustained efforts have been made to provide care to the hypertensive population since the 1980s [5] through its Mixed Healthcare system (public and private), with 85% of the population using public-health insurance and services in 2015 [6]. Since the 1980s, two major health system interventions were introduced to improve the management of hypertension. First, in 2002, the former hypertension disease specific programme in primary public care was transformed into an integrated risk-stratified based model: the “Cardiovascular Health Program” and second, in 2005, a law was passed (Garantías Explícitas en Salud [GES]) which warranted timely access and financial coverage (e.g. medicines free-of-charge) to all insured Chileans (public and private) for the most prevalent chronic diseases, including hypertension [7]. These efforts are aligned with the current health goals for 2010 to 2020 of increasing the level of controlled hypertension (BP < 140/90 mmHg) in relative terms by 50% [8].

While hypertension prevalence (defined as BP ≥ 140/90 mmHg or use of antihypertensive treatment) across 90 countries remained stable between 2000 and 2010 at around 30%, key hypertension care cascade indicators - awareness, treatment and control – increased slightly from 41 to 47%; 32 to 37%; and 34 to 37% respectively [2]. Despite the availability of highly effective antihypertensive medications, the majority of hypertensive patients on treatment do not therefore achieve BP control [2].

Hypertension prevalence, and levels of uncontrolled hypertension, would be even higher if a stricter definition of hypertension based on lower thresholds for defining elevated BP is adopted. According to the 2017 American College of Cardiology/American Heart Association guidelines (2017 ACC/AHA) [9], the commonly used (seventh Joint National Committee [JNC] 7) [10] threshold for high BP (BP ≥ 140/90 mmHg) should be lowered to BP ≥ 130/80 mmHg. This change in criteria calls for a more aggressive approach in order to decrease the risk of CVD events occurring at lower levels of BP. The recommendation to lower BP thresholds was prompted by the findings of SPRINT (Systolic Blood Pressure Intervention Trial) which found significant reductions in the primary composite outcome (myocardial infarction, other acute coronary syndromes, stroke, heart failure, or death from cardiovascular causes) and in overall mortality among adults in the intensive treatment group (BP target < 120 mmHg) versus those in the standard treatment group (BP target < 140 mmHg) [11].

Attainment of care cascade indicators for various diseases have been recently described for LMICs [12] and for HICs [13] using cross-sectional nationally representative health examination surveys, based on the JNC 7 definition of hypertension (BP ≥ 140/90 mmHg or use of antihypertensive treatment). Geldsetzer et al. showed a prevalence of hypertension of around 30% for most Latin American and Caribbean countries, with the lowest prevalence in Ecuador (9%) and the highest in Grenada (41%); however, levels were not directly comparable due to differences in age [12]. Zhou B et al. showed higher levels of hypertension for HICs than for most LMICs, with values amongst those aged 40–79 years ranging between 33% in Australia to over 52% in Finland [13].

Recent cross-sectional analysis of Latin American data collected as part of the Prospective Urban Rural Epidemiology (PURE) study was based on 33,276 participants aged 35–70 years from urban and rural communities in six countries (Argentina, Brazil, Chile, Colombia, Peru and Uruguay). The study by Lemelas et al. reported significant variations in prevalence (18–52%), awareness (52–65%), treatment (47–63%) and control (16–30%) [14]. However, the analytical sample was not nationally representative, and its cross-sectional design meant that no evidence could be provided on trends in these indicators over time.

There is scarce evidence of recent trends in the hypertension care cascade from countries such as Chile that have experienced fast epidemiologic transitions. To the best of our knowledge, no studies to date in the Latin American and Caribbean region (LAC) have quantified these trends using nationally representative, measured BP data, and only one study (in Peru) has assessed the implications of lowering the BP thresholds in line with the 2017 ACC/AHA guidelines (albeit using an indicator of high BP alone) [15, 16]. Using data from three nationally representative Chilean health examination surveys covering a 15-year period (2003; 2010; 2017), the aims of the present study were to: (1) quantify trends in hypertension prevalence and in attainment at each step of its care cascade (i.e. awareness, treatment and control), including an assessment of gender disparities; and (2) quantify the impact of lowering the BP thresholds on these indicators.

## Methods

### Study design and setting

We used data from the three most recent Chilean National Health Surveys (Encuesta Nacional de Salud, ENS: 2003; 2010; 2017). The 2003 ENS sample was stratified with a random sub-sample selected from participants of the Quality of Life and Health Survey [17]. The sampling frame for the Quality of Life and Health Survey was household information from the National Census of 1992, and sampling was carried out by a stratified, cluster design. The subsample of participants for the ENS 2003 was selected using the same age-gender-region structure of the original sample frame, except for the oversampling of one region. Fresh samples were selected for the 2010 and 2017 surveys based on stratified cluster sampling. Sampling for both was based on the master sample frame of the Chilean National Institute of Statistics and the Population and Housing Census of Chile.

The target population for each survey was the free-living general population aged 17 years or older (2003) and aged 15 years or older (2010 and 2017). Institutionalised and non-Spanish speaking individuals were excluded. Persons aged 65 years or over were oversampled. One eligible person was randomly selected for interview within the selected household using a Kish grid [18]. ENS survey instruments and protocols are described in detail elsewhere [19].

### Ethics approval and consent to participate

The study protocol and ethical consent forms were approved by the ethics committee of the Pontificia Universidad Católica de Chile (PUC) and the Chilean Ministry of Health (ENS 2003: number could not be retrieved; 2010: 09–113; 2017: 16–019). Persons selected for inclusion provided informed and signed consent before participation.

### Data collection

Data collection procedures were generally similar across the three surveys. In the first home visit, a trained interviewer applied health questionnaires face-to-face, including the following questions regarding hypertension awareness: “*Have you ever been told by a doctor, nurse or health care provider that you have high blood pressure?*” and hypertension treatment (regardless of awareness) “*Are you currently carrying or doing any program or treatment indicated by a health professional to keep your blood pressure under control?”*. Participants reporting that they were on treatment were asked about type of treatment (response options: *medications, treatment without medication, or both).* During the second visit, a trained nurse measured BP and recorded the medications participants were currently using (prescribed or not) via a detailed inventory. Medications were classified using the anatomical therapeutic chemical (ATC) classification system [20]. Sitting BP was measured after a five-minute rest using an upper arm monitor (Omron, Healthcare Co Ltd., Kyoto, Japan: models HEM713C, HEM742 and HEM7200 in 2003, 2010 and 2017, respectively) with appropriately sized arm cuffs, with a two-minute pause between readings. Two BP readings were taken in 2003 while three readings were taken in 2010 and 2017. To ensure like-for-like comparisons, we used the average of the first- and second-readings in each year. We decided at the outset of the present study that this approach would provide the most accurate estimate of change in hypertension across the three surveys giving more value to the reproducibility of the results to estimate trends over time [21].

### Definitions of hypertension and the care cascade

Estimates of hypertension prevalence vary by choice of high BP cut-points [9]. We compared two different definitions of hypertension. First, we identified participants with hypertension based on the seventh report of the JNC on prevention, detection, evaluation and treatment of high blood pressure: SBP/DBP ≥140/90 mmHg or current use of antihypertensive treatment (hereafter referred to as the JNC 7 guideline) [10]. Second, we identified participants with hypertension based on the 2017 ACC/AHA guidelines: SBP/DBP ≥130/80 mmHg or current use of antihypertensive treatment [9].

We focused on three steps of the hypertension care cascade: awareness, treatment, and control. Among those classified as hypertensive, we defined: (1) awareness as the report of prior diagnosis of high BP by a healthcare professional; (2) treatment (in our main analyses) as the current use of antihypertensive medication as identified in the medicine inventory (ATC codes: C02, C03, C07, C08, C09); and (3) control according to the JNC 7 (BP < 140/90 mmHg) and the 2017 ACC/AHA (BP < 130/80 mmHg) guidelines.

### Sociodemographic characteristics

Participants were grouped into six age categories (17–34; 35–44; 45–54; 55–64; 65–74; 75+). Educational status based on years of formal education was grouped as low (< 8 years); medium (8–12 years) and high (> 12 years); place of residence was grouped as urban or rural.

### Statistical analysis

Analyses were restricted to adults aged 17 years or over to ensure comparability across the three surveys. First, we summarised the sociodemographic profile (age; gender; educational level; place of residence) and estimated average SBP/DBP levels in each survey amongst all participants with valid BP and medicine data.

Second, amongst those classified as hypertensive (JNC 7 guideline), we calculated the levels of awareness, treatment and control. Third, pooling data across years, we used age-adjusted logistic regression to estimate the gender-specific trends in hypertension (JNC 7 guideline). Among those classified as hypertensive, we used age-adjusted logistic regression to calculate gender-specific trends in awareness, treatment, and control. In each analysis survey year and age were entered into the models as a three-category independent variable and as a single continuous variable, respectively. Results were summarised using Odds Ratios (ORs) with accompanying 95% confidence intervals (95% CI). Pairwise comparisons were used to evaluate change over time (i.e. 2010 versus 2003; 2017 versus 2003; and 2017 versus 2010). Age-adjusted linear regression models were used to test for significant trends in mean SBP and DBP (regardless of treatment and separately by treatment status).

Fourth, we quantified the difference in hypertension prevalence and in levels of awareness, treatment and control between the current (JNC 7) and new (2017 ACC/AHA) guidelines. For each survey year, participants were classified into one of four mutually exclusive groups. According to the JNC 7 guidelines, these groups were defined as follows: normotensive (< 140/90 mmHg); treated and controlled (< 140/90 mmHg); treated, but uncontrolled (≥140/90 mmHg); and untreated and uncontrolled (≥140/90 mmHg). The corresponding classification using the 2017 ACC/AHA guidelines was as follows: normotensive (< 130/80 mmHg); treated and controlled (< 130/80 mmHg); treated, but uncontrolled (≥130/80 mmHg), and untreated and uncontrolled (≥130/80 mmHg). Applying the 2017 Chilean census data to the ENS 2017 [22], we estimated the number of additional adults who would be eligible for antihypertensive treatment based on the 2017 ACC/AHA guidelines.

### Sensitivity analysis

In our main analysis, ascertaining use of antihypertensive treatment through ATC codes may slightly overestimate prevalence as some medicines can be used for other conditions without the co-existence of hypertension. To determine the robustness of our ascertainment of antihypertensive treatment, we estimated hypertension prevalence based on the JNC 7 guidelines using self-reported treatment and compared the difference between the two sets of estimates (i.e. self-report versus ATC codes collected via the detailed medicine inventory). In a second sensitivity analysis we estimated the change in hypertension prevalence using the average of the second and third BP readings in the 2010 and 2017 surveys.

Analyses were based on complete-cases and were weighted accounting for differences in selection probability (e.g. selection of one person per household) and non-response rates. *P*-values < 0.05 were classified as statistically significant (two-tailed). All analyses were conducted in Stata V14.0 (StataCorp LP, College Station, Texas, U.S.) adjusting for the complex survey design.

## Results

### Sample characteristics

Response rates for the total sample were 63, 75 and 67% in 2003, 2010 and 2017 respectively. Overall, 3614, 5267 and 6074 adults took part in the ENS 2003, 2010, and 2017 surveys, respectively. Of these, 3448 (95%), 4863 (92%) and 5379 (86%) adults took part in the nurse visit; the vast majority of these (99%) had valid BP and medicine data. Table 1 shows the sociodemographic profile and average levels of BP in each survey year amongst the 13,605 participants aged 17 years or over with valid BP and medicine data. Characteristics were similar across the three surveys, with the exception of an increase over time in the proportion of participants in the highest educational group (> 12 years of formal education).

**Table 1:** Sociodemographic characteristics by survey year. Chile ENS2003–2010-2017

		ENS 2003		ENS 2010		ENS 2017	
		n^a^	% or mean (SE)	n^a^	% or mean (SE)	n^a^	% or mean (SE)
Sample with valid BP and medicine data		3416		4820		5369	
Gender	Male	1558	48.9	1919	48.4	1945	48.9
	Female	1858	51.1	2901	51.6	3424	51.1
Age %	17-34y	863	40.7	1361	35.5	1342	33.9
	35-44y	583	21.6	874	22.5	813	20.1
	45-54y	608	16	892	16.8	879	14.7
	55-64y	486	10.8	749	13	978	17.3
	65-74y	527	6.8	536	7.4	786	8
	75y+	349	4.2	408	4.7	571	5.9
Educational level %	Low (<8y)	1362	25.4	1277	19.1	1319	16.9
	Medium (8-12y)	1625	55.5	2531	55.5	2808	54.1
	High(>12y)	421	19.1	912	25.3	1195	29
Place of residence	Urban	2798	86.4	4099	86.9	4513	89.2
	Rural	618	13.6	721	13.1	856	10.8
SBP mmHg		3416	127.8 (0.52)	4820	126.9 (0.48)	5369	124.9 (0.46)
DBP mmHg		3416	79.9 (0.34)	4820	76.6 (0.27)	5369	74.7 (0.25)

Estimates are weighted for the complex survey design

*ENS* Encuesta Nacional de Salud, Chilean National Health Survey, *BP* Blood pressure, *SBP* Systolic BP, *DBP* Diastolic BP, *SE* Standard error

^a^Unweighted sample size. Column %‘s shown for sociodemographic characteristics

### Hypertension and its care cascade (awareness, treatment and control)

**Fig. 1:**
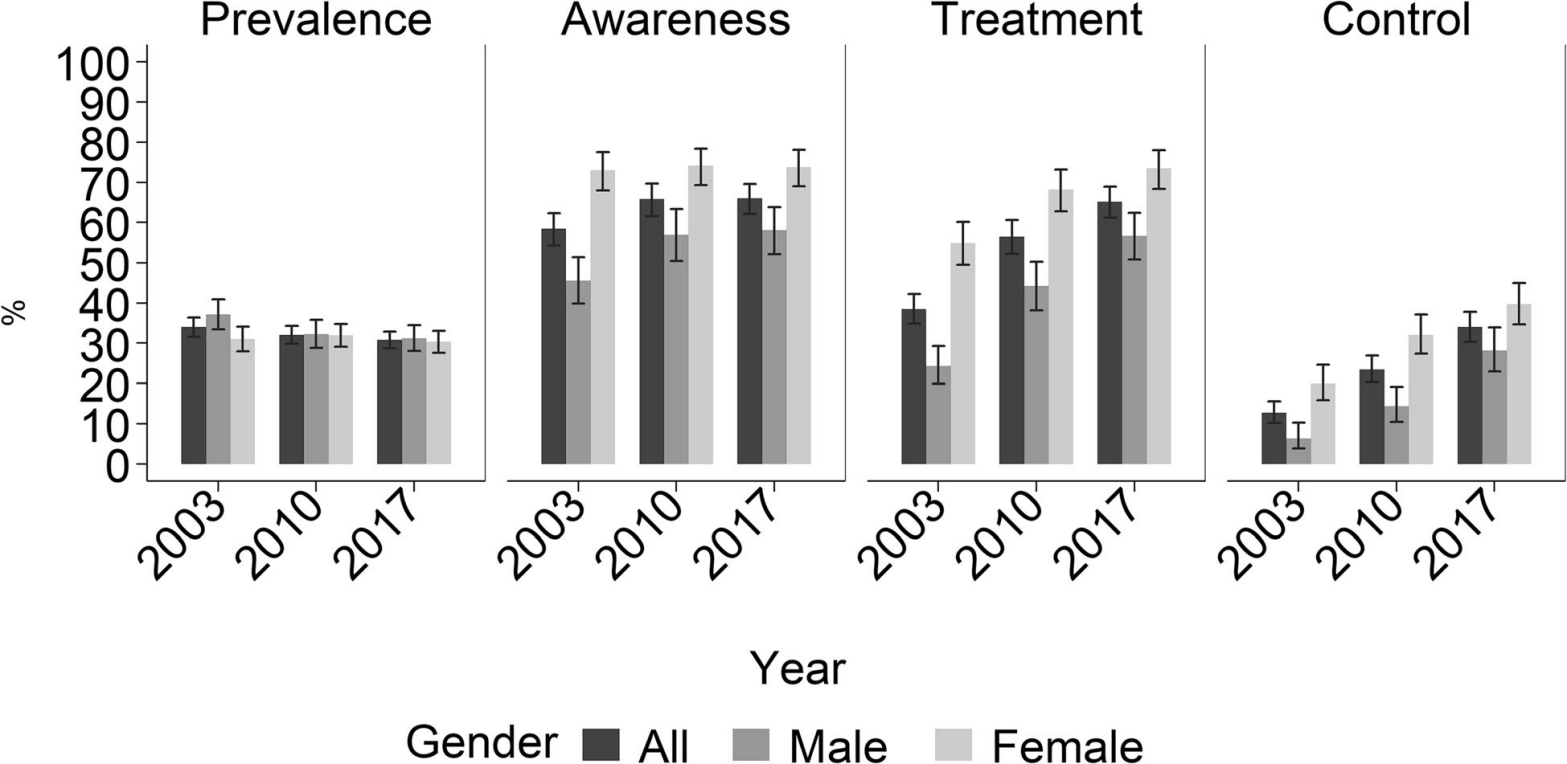
Hypertension prevalence, awareness, treatment and control by gender. Chile ENS2003–2010-2017. Definitions according to the JNC 7 guideline. Prevalence: SBP/DBP ≥140/90 mmHg or current use of antihypertensive treatment; Awareness: prior diagnosis of high blood pressure; Treatment: current use of antihypertensive medication according to ATC codes; and Control: SBP/DBP < 140/90 mmHg. Levels of hypertension estimated among all adults. Levels of awareness, treatment and control estimated amongst those classified as hypertensive

Based on the (current) JNC 7 guidelines (SBP/DBP ≥140/90 mmHg or current use of antihypertensive treatment), Fig. 1 shows the levels of hypertension and levels of attainment at each care cascade step (awareness, treatment, and control) across the three surveys. The estimates and accompanying 95% confidence intervals (95% CI) are provided as supplementary data (Additional file 1: Table S1). For brevity, we report here on the change between the first- and last-surveys (i.e. 2003 and 2017). Among all adults, hypertension prevalence decreased slightly from 34.0% (95% CI: 31.6–36.4%) to 30.8% (95% CI: 28.7–32.9%). Hypertension prevalence decreased among males from 37.1% (95% CI: 33.5–40.9%) to 31.2% (95% CI: 28.1–34.5%) and decreased among females from 31.0% (95% CI: 28.0–34.1%) to 30.3% (95% CI: 27.6–33.1%).

Among those classified as hypertensive, attainment at each cascade step mainly showed improvement. Levels of treated- and controlled-hypertension were significantly higher in 2017 than in 2003 (65% versus 41% for treatment, *P* < 0.001; 34% versus 14% for control, *P* < 0.001), while levels of awareness were stable (66% versus 59%, *P* = 0.130) (Fig. 1; Additional file 1: Table S1). Trends were similar by gender. First, levels of awareness among males increased from 45.6% (95% CI: 39.9–51.4%) to 58.1% (95% CI: 52.2–63.8%); levels were higher among females but remained stable at around 73%. Second, levels of treatment among males increased from 24.3% (95% CI: 19.9–29.3%) to 56.7% (95% CI: 50.9–62.4%) and increased among females from 54.9% (95% CI: 49.5–60.1%) to 73.5% (95% CI: 68.4–78.0%). Third, levels of controlled hypertension among males increased from 6.3% (95% CI: 3.8–10.3%) to 28.2% (95% CI: 23.0–33.9%) and increased among females from 19.9% (95% CI: 15.9–24.7%) to 39.7% (95% CI: 34.7–45.0%).

### Age-adjusted trends in hypertension and its cascade of care

**Fig. 2:**
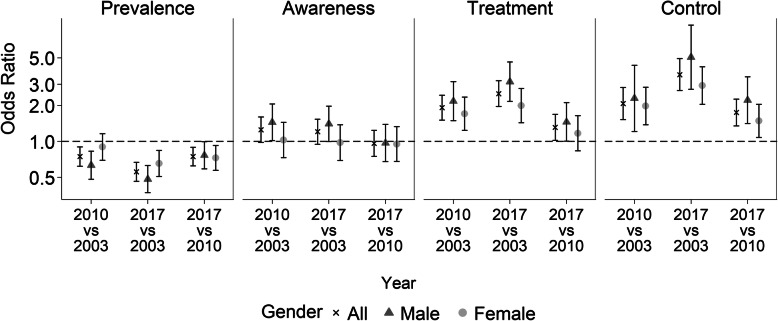
Trends for hypertension prevalence, awareness, treatment and control by gender. Chile ENS2003–2010-2017. Odds ratios from age-adjusted logistic regression. Definitions according to the JNC 7 guideline. Prevalence: SBP/DBP ≥140/90 mmHg or current use of antihypertensive treatment; Awareness: prior diagnosis of high blood pressure; Treatment: current use of antihypertensive medication according to ATC codes; and Control: SBP/DBP < 140/90 mmHg. Odds of hypertension estimated among all adults. Odds of awareness, treatment and control estimated amongst those classified as hypertensive

Figure 2 shows the age-adjusted trends in hypertension and in attainment at each cascade step by gender based on logistic regression models. Estimates and accompanying 95% CIs are provided as supplementary data (Additional file 1: Table S2). Among males, the odds of hypertension decreased significantly between 2003 and 2010 (OR: 0.63; 95% CI: 0.48–0.83) and between 2010 and 2017 (OR: 0.77; 95% CI: 0.59–1.00). The odds of hypertension decreased for females between 2010 and 2017 (OR: 0.73; 95% CI: 0.57–0.92). In a pooled analysis, the odds of hypertension were significantly lower for females than for males (OR: 0.73; 95% CI: 0.64–0.84, *P* < 0.001) (Additional file 1: Table S3).

Amongst those classified as hypertensive, the odds of awareness did not change significantly over time, except among males, where the odds increased from 2003 to 2010 (OR: 1.45; 95% CI: 1.02–2.06). Compared with 2003, the odds of treatment and control were significantly higher in 2010 and in 2017 for both genders (Fig. 2; Additional file 1: Table S2). In a pooled analysis, the age-adjusted odds of awareness (OR: 2.27; 95% CI: 1.86–2.77, *P* < 0.001), treatment (OR: 2.53; 95% CI: 2.07–3.09, *P* < 0.001) and control (OR: 3.53; 95% CI: 2.63–4.73, *P* < 0.001) were significantly higher for females than for males (Additional file 1: Table S3).

### Age-adjusted trends in SBP and DBP

Average levels of age-adjusted SBP and DBP among all adults (i.e. regardless of treatment) decreased significantly over the 15-year period for both genders (Additional file 1: Table S4). For example, mean SBP decreased by 4.4 mmHg (95% CI: 2.9–6.0 mmHg) and by 5.8 mmHg (95% CI: 4.3–7.4 mmHg) between 2003 and 2017 among males and females, respectively.

Additional analyses stratified by treatment status showed that mean BP levels decreased significantly among all groups, with the exception of no significant change in mean SBP among females using antihypertensive treatment (Additional file 1: Table S4).

### Hypertension prevalence based on lower BP thresholds

**Fig. 3:**
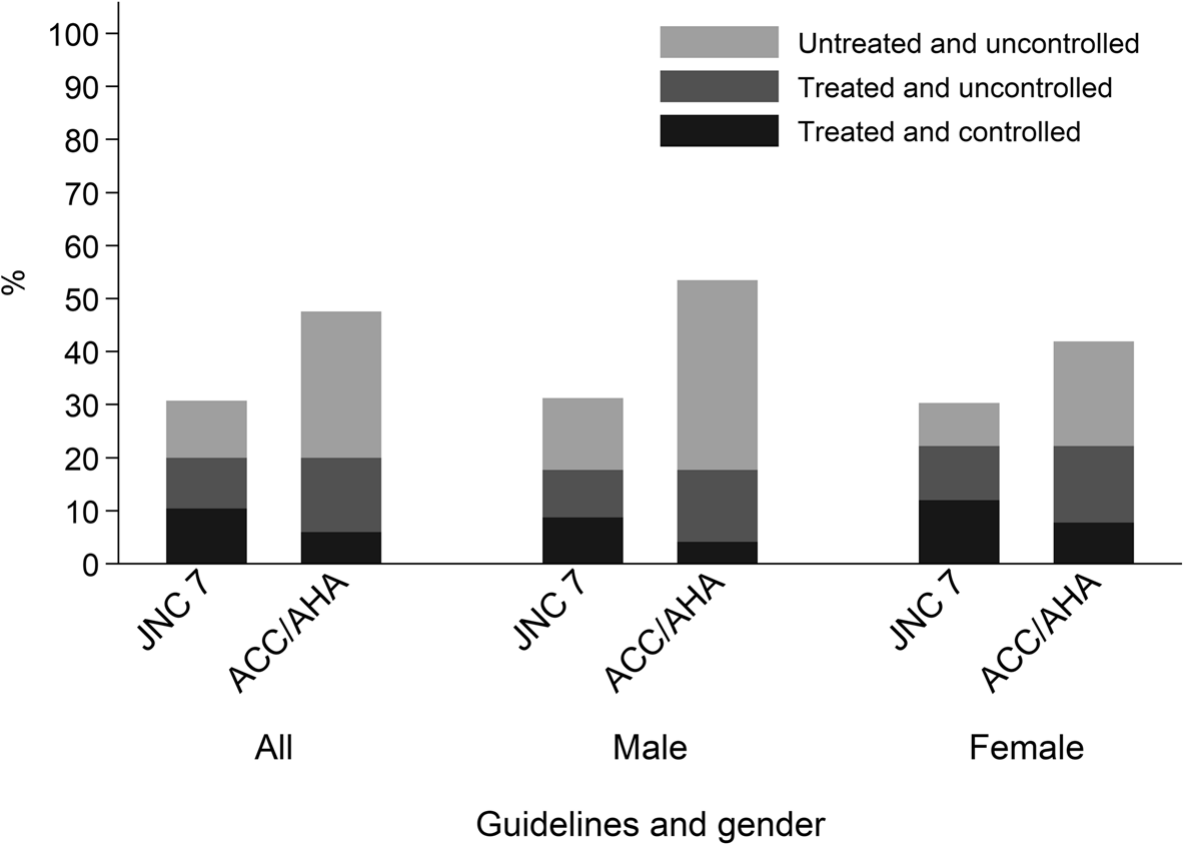
Prevalence, treatment and control of hypertension according to JNC 7 and 2017 ACC/AHA guidelines by gender. Chile ENS2017. JNC 7 groups defined as follows: normotensive (< 140/90 mmHg); treated and controlled (< 140/90 mmHg); treated and uncontrolled (≥140/90 mmHg); untreated and uncontrolled (≥140/90 mmHg). 2017 ACC/AHA groups defined as follows: normotensive (< 130/80 mmHg); treated and controlled (< 130/80 mmHg); treated and uncontrolled (≥130/80 mmHg); untreated and uncontrolled (≥130/80 mmHg). Use of antihypertensive treatment ascertained using ATC codes

Based on the ENS 2017 data, Fig. 3 shows the difference in hypertension prevalence and in the proportion of adults attaining each step of the care cascade based on the new (2017 ACC/AHA) and current (JNC 7) guidelines. Estimates and accompanying 95% CIs are provided as supplementary data (Additional file 1: Table S5). Overall, hypertension prevalence in 2017 would be about 17% percentage points higher in absolute terms if the BP threshold was lowered to < 130/80 mmHg (2017 ACC/AHA: 47.6, 95% CI: 45.2–50.0%; JNC 7: 30.7, 95% CI: 28.7–32.9%); a relative increase of around 55%. Based on the 2017 census, we estimate that an additional 2.3 million adults aged 17 years or over would therefore be classified as hypertensive and so be eligible for antihypertensive treatment. We estimate that the proportion of adults in the population with uncontrolled and untreated hypertension in 2017 using the 2017 ACC/AHA guideline would be about 17% percentage points higher (2017 ACC/AHA: 27.5%; 95% CI: 25.4–29.8%; JNC 7: 10.7%; 95% CI: 9.4–12.2%); whilst the proportion of adults with treated, but uncontrolled hypertension would be about 4.4% percentage points higher (2017 ACC/AHA: 14.0%; 95% CI: 12.6–15.5%; JNC 7: 9.6%; 95% CI: 8.5–10.8%).

### Sensitivity analysis

Our sensitivity analysis showed similar levels of hypertension (based on the JNC 7 definition), whether using ATC-codes from the detailed nurse-administered medicine inventory or self-reported data to ascertain use of antihypertensive treatment. The absolute difference in the two sets of estimates (ATC versus self-reported) was 0.1, 3.0 and 1.2% in 2003, 2010 and 2017 respectively (Additional file 1: Figure S1). Repeating our main analyses using the average of the second- and third-BP readings in ENS 2010 and ENS 2017 produced similar evidence regarding trends over time. For example, the estimated OR for hypertension prevalence in 2017 versus 2003 slightly decreased from 0.56 (95% CI: 0.46–0.67) to 0.53 (95% CI: 0.44–0.63). The OR for awareness slightly increased from 1.20 (95% CI: 0.94–1.53) to 1.27 (95% CI: 0.99–1.63). The pattern was similar for treated- and controlled-hypertension. The OR for treatment increased from 2.49 (95% CI: 1.95–3.20) to 2.73 (95% CI: 2.11–3.52); the OR for control increased from 6.85 (4.69–10.02) to 7.58 (5.18–11.08) (Additional file 1: Table S3).

## Discussion

Data on current trends in hypertension prevalence and changes over time in the levels of attainment at each step of its care cascade (i.e. awareness, treatment and control) are required in the LAC region. Our analysis of Chilean health examination survey data indicates that around three-in-ten adults aged 17 years or older had hypertension (according to the JNC 7 guideline: BP ≥ 140/90 mmHg or use of antihypertensive treatment), with the prevalence from 2003 to 2017 showing a small but significant decline (from 37 to 31% for males; from 31 to 30% for females). Mean SBP and DBP levels also decreased at the population level over the 15-year period.

### Explanations for observed trends

Average BP levels fell steadily worldwide between 1975 and 2015 [1]. Some uncertainty exists about the drivers of the worldwide trends in average BP and hypertension prevalence, particularly as the reductions in BP levels have been accompanied by increases in a number of the leading risk factors for high BP, including high body mass index (BMI) and diabetes [23]. Our results also showed decreases in average BP levels regardless of treatment status (with the exception of no significant change in mean SBP among females on treatment), while evidence from other studies analysing Chilean data over the same time period shows that levels of obesity have increased, whilst levels of physical inactivity were unchanged [24]. The decrease in BP can be explained at least partially by the increased detection of high BP by health care professionals (awareness) and by the subsequent wider uptake of antihypertensive treatments [1, 25]. Evidence consistently shows that higher levels of hypertension are associated with lower levels of income and formal education [26, 27]. Therefore, some of the BP decline in Chile could be attributed to the decrease in absolute poverty from 29 to 9% and the increase in the average length of time spent in formal education from 10 to 11 years between 2009 and 2017 [28]. The decline in hypertension prevalence in Chile was similar to that observed in other HICs, potentially driven by the decrease in risk factors which buffered the expected increase due to the rises in obesity and diabetes. For instance, salt intake [29] and exposure to dietary trans fatty acids and smoking decreased over time [24, 30]. Since 2011, the average salt levels in bread has gradually reduced and stop signs were introduced in 2012 on packaged foods “high in” energy, sodium, sugars, and saturated fats, which contributed to a healthier food industry reformulation [31, 32]. Despite the progress over the 15-year period, the prevalence of hypertension (defined as BP ≥ 140/90 mmHg or use of antihypertensive treatment) is currently slightly higher in Chile compared to that found in HICs [13].

According to our results, current levels of attainment at each step of the hypertension care cascade are higher in Chile compared to most LMICs [12], while compared to HICs [13], levels of awareness, treatment and control were lower, higher and similar respectively. We found that levels of treated and controlled hypertension significantly increased from 2003 to 2017, while levels of awareness increased only among males between 2003 and 2010. In agreement with global trends, our analyses showed that the proportions of adults reaching each stage of the care cascade were similar to those reported in other HICs and were higher than those in LMICs between 2000 and 2010 [2, 12]. Several explanations have been put forward for the global improvement in levels of hypertension awareness, treatment and control, including increases in BP screening at the primary care and community levels, securing better treatment availability, reducing treatment costs, improving treatment adherence and preventing clinical inertia [33]. The GES was launched in Chile in 2005 with a wide marketing strategy and helped to disseminate evidence-based guidelines with simplified recommendations nationwide. In 2014, the law was enforced with an additional regulation called FOFAR, which, for the publicly insured, warranted medicines free-of-charge for hypertension, diabetes and dyslipidaemia. Although we cannot directly assess the impact of these programmes with the ENS data, we can speculate that they have had at least some positive impact through the improvements in treatment and control levels presented here.

### Evidence on gender disparities

Our analyses show no significant difference in hypertension prevalence by gender. However, levels of attainment at each cascade step were higher among females. These gender disparities were also reported among LMICs [12] and HICs [2, 13]. However, the gender gap was wider in Chile than in other HICs. For example, current levels of controlled hypertension were 41 and 26% higher in relative terms among females in Chile (according to our results) and in HICs, respectively [2]. Potentially these gender disparities arise from higher levels of health care services utilisation among females and from lower long-term adherence to antihypertensive treatment among males [34]. Although gender disparities exist, the trends show some evidence of faster improvements among males. Our analyses show that the Chilean 2010 to 2020 health goal of increasing the level of controlled hypertension by 50% in relative terms has been achieved among males, but is only at the halfway point among females since the relative increases from 2010 to 2017 were 97 and 24% for males and females respectively [8].

### Implications of lowering BP thresholds

Using the 2017 data we found that implementing the 2017 ACC/AHA guideline – i.e. lowering the BP threshold from 140/90 mmHg to 130/80 mmHg - would result in 2.3 million more Chilean adults being classified as hypertensive and so be eligible for antihypertensive treatment. This is in addition to the 1.5 million hypertensive adults currently untreated according to the current (JNC 7) guideline. The relative increase of about 55% in hypertension prevalence as a result of adopting the 2017 ACC/AHA guideline seems to be higher in Chile than those estimated (using similar methods) in the United States (27%), China (45%), Spain (42%) [15, 16], Venezuela (25%) and Colombia (34%) [35], but lower than in Peru (130%) [36]. The definitions used in Venezuela, Colombia and Peru were based on high BP alone [35, 36], and so is not strictly comparable with our findings. This new scenario would be a massive challenge for Chile, requiring significant increases in public health expenditure, especially for health-care services and medicines. Implementing the new guideline would potentially increase the absolute number of hypertensives who are aware and on treatment over time. There is a growing debate about the merits of lowering the BP threshold, including concerns about the expected costs of implementation [35, 37].

### Strengths and limitations

One strength of our study is the use of nationally representative health examination survey data, in contrast to the Prospective Urban Rural Epidemiology (PURE) study which covers only a few cities [14], and our use of objective measures which overcome the limitations of self-report data. However, our study has a number of limitations. First, to ensure comparability across the three surveys, we used the average of the first and second BP readings. Compared to using the average of the second- and third-readings, our approach could have slightly (< 1%) overestimated hypertension prevalence and underestimated levels of controlled hypertension [38]. Reassuringly, our findings were similar when we repeated our main analyses by taking the average of the second and third readings in the 2010 and 2017 surveys. Second, according to the JNC 7 and 2017 ACC/AHA guidelines, the diagnosis of hypertension should be made at follow-up visits [9, 10]. Evaluation of BP during a single visit (as done in the ENS surveys) may overestimate the true prevalence as raised BP is not necessarily persistent. According to analyses of Chilean data, a small but statistically significant reduction in hypertension prevalence (1%) was found when BP measurement was repeated in a follow-up visit [39]. Moreover, the impact of the ‘white coat effect’ (i.e. transient increase in BP produced by the presence of a healthcare professional) or of ‘masked hypertension’ (i.e. non-elevated BP in clinical but elevated in ambulatory monitoring) on levels of hypertension could not be estimated in our study.

Third, recall bias could also have impacted on our estimated levels of awareness and treatment. Ascertaining use of antihypertensive treatment based on ATC codes from the medicine inventory could have produced a slight overestimation of prevalence since some medicines can be used for other conditions without the co-existence of hypertension. However, our sensitivity analysis showed that the magnitude of the bias was very small in 2003 and 2017 and only slightly higher in 2010. This suggests a minor recall bias in participants’ self-reports or low use of antihypertensive treatments for conditions other than hypertension. The reason behind the slighly higher bias seen in 2010 could be a slightly modification in the question phrasing for ascertaining use of antihypertensive treatment [19]. Fourth, although the same BP monitor was used in each survey, use of different models may have weakened comparability to some extent. Finally, as in other nationally representative health examination surveys, levels of response to the Chilean health survey have decreased over time. However, the current levels of response are comparable to those achieved by other national health examination surveys [40].

Response rates to the surveys were 63, 75 and 67% for 2003, 2010 and 2017 respectively. The smaller sample size for the 2017 survey resulted in lower precision for the survey estimates and lower statistical power for detecting change over time. Our analyses show that the proportion of female and younger participants in the analytical samples increased over time (data not shown). However, the impact of any differential non-response over time would be reduced by our decision to stratify the analyses by age and gender and by use of non-response weighting. It is also worth highlighting the conclusions of previous studies which indicate that the overall response rate for a survey, in and of itself, is not a good indicator of the level of non-response bias [41, 42].

## Conclusions

In conclusion, mean levels of BP in the untreated and treated populations have declined in Chile during the last 15 years (with the exception of no significant change in mean SBP among females on treatment), while levels of treatment and control among adults with hypertension have increased. The introduction of Universal Access to care for hypertension in 2005 may have accounted at least partly for the rise in levels of treatment and control since 2003. Nevertheless, more population-based interventions (such as increasing taxes on unhealthy foods) and individual level interventions (such as wider use of BP screening among younger males) that address both behavioural risk and protective factors are needed to increase levels of controlled hypertension. Regardless of the hypertension definition, innovative and collaborative strategies are needed to improve levels of attainment at each step of the hypertension care cascade, including the promotion of screening and access to care, together with interventions to increase treatment coverage and its adherence, especially among males and high CVD risk populations.

## Supplementary information

**Additional file 1: Table S1.** Hypertension prevalence, awareness, treatment and control. Chile ENS2003–2010-2017. **Table S2.** Trends for hypertension prevalence, awareness, treatment and control. Chile ENS2003–2010-2017. **Table S3.** Trends for hypertension prevalence, awareness, treatment and control according to the definition of mean systolic and diastolic blood pressure (BP). Chile ENS2003–2010-2017. **Figure S1.** Prevalence of hypertension (BP ≥ 140/90 mmHg or use of antihypertensive treatment) using to define treatment self-reported data (SR) or Anatomical Therapeutic Chemical (ATC) codes from the medicine inventory. **Table S4.** Blood pressure trends according to treatment status. Chile ENS2003–2010-2017. **Table S5.** Prevalence, treatment and control of hypertension according to JNC 7 and 2017 ACC/AHA guidelines. Chile ENS2017.

## Data Availability

The datasets generated and/or analysed during the current study are available in the Chilean Ministry of Health webpage: Departamento de Epidemiología, Ministerio de Salud de Chile, Encuesta Nacional de Salud, 2016–2017 at http://epi.minsal.cl/bases-de-datos/

## Abbreviations

ACC: American College of Cardiology
AHA: American Heart Association
ATC: Anatomical therapeutic chemical
BP: Blood pressure
CVD: Cardiovascular disease
DBP: Diastolic blood pressure
ENS: Encuesta Nacional de Salud (Chilean health survey)
GES: Garantías Explícitas en Salud
HICs: High-income countries
JNC 7: Seventh report of the Joint National Committee on Prevention, Detection, Evaluation, and Treatment of High Blood Pressure
LAC: Latin America and Caribbean region
LMICs: Low- and middle- income countries
PURE: Prospective Urban Rural Epidemiology study
SBP: Systolic blood pressure
