# Supplementary material for: Hypertension care cascade in Chile: a serial cross-sectional study of national health surveys 2003-2010-2017
Source: BMC Public Health. 2020 Sep 14;20:1397. doi: 10.1186/s12889-020-09483-x (PMC7490861; doi:10.1186/s12889-020-09483-x)
Supplement: Supplementary file 1 — Additional file 1: Table S1. Hypertension prevalence, awareness, treatment and control. Chile ENS2003–2010-2017. Table S2. Trends for hypertension prevalence, awareness, treatment and control. Chile ENS2003–2010-2017. Table S3. Trends for hypertension prevalence, awareness, treatment and control according to the definition of mean systolic and diastolic blood pressure (BP). Chile ENS2003–2010-2017. Figure S1. Prevalence of hypertension (BP ≥ 140/90 mmHg or use of antihypertensive treatment) using to define treatment self-reported data (SR) or Anatomical Therapeutic Chemical (ATC) codes from the medicine inventory. Table S4. Blood pressure trends according to treatment status. Chile ENS2003–2010-2017. Table S5. Prevalence, treatment and control of hypertension according to JNC 7 and 2017 ACC/AHA guidelines. Chile ENS2017. [file 12889_2020_9483_MOESM1_ESM.docx]

**Supplemental Digital Content for:**

**Hypertension care cascade in Chile: a serial cross-sectional study of national health surveys 2003-2010-2017.**

PASSI-SOLAR Alvaro^a,b,*^, MARGOZZINI Paula^b^, MINDELL Jennifer S^a^, RUIZ Milagros^a^, VALENCIA-HERNANDEZ Carlos A^a^, SCHOLES Shaun^a^

^a^ Department of Epidemiology and Public Health, University College London, 1‐19 Torrington Place, London, WC1E 6BT, UK. ^b^ Department of Public Health, School of Medicine, Pontificia Universidad Católica de Chile, Diagonal Paraguay 362, CP 88330077, Santiago, Chile.

**Table of contents**

[Table S1. Hypertension prevalence, awareness, treatment and control. Chile ENS2003-2010-2017 2](#_Toc42013360)

[Table S2. Trends for hypertension prevalence, awareness, treatment and control. Chile ENS2003-2010-2017 3](#_Toc42013361)

[Table S3. Trends for hypertension prevalence, awareness, treatment and control according to the definition of mean systolic and diastolic blood pressure (BP). Chile ENS2003-2010-2017 4](#_Toc42013362)

[Figure S1. Prevalence of hypertension (BP≥140/90 mmHg or use of antihypertensive treatment) using to define treatment self-reported data (SR) or Anatomical Therapeutic Chemical (ATC) codes from the medicine inventory. Chile ENS2003-2010-2017 5](#_Toc42013363)

[Table S4. Blood pressure trends according to treatment status. Chile ENS2003-2010-2017 6](#_Toc42013364)

[Table S5. Prevalence, treatment and control of hypertension according to JNC 7 and 2017 ACC/AHA guidelines. Chile ENS2017 7](#_Toc42013365)

### **Table S1. Hypertension prevalence, awareness, treatment and control. Chile ENS2003-2010-2017**

| **Population** | **Year** | **Prevalence** | | | **Awareness** | | | **Treatment** | | | **Control** | | |
| --- | --- | --- | --- | --- | --- | --- | --- | --- | --- | --- | --- | --- | --- |
|  |  | **%** | **95% CI** | | **%** | **95% CI** | | **%** | **95% CI** | | **%** | **95% CI** | |
| **All** | 2003 | 34.0 | 31.6 | 36.4 | 58.4 | 54.4 | 62.3 | 38.5 | 34.9 | 42.3 | 12.7 | 10.2 | 15.6 |
|  | 2010 | 32.0 | 29.9 | 34.3 | 65.8 | 61.6 | 69.7 | 56.5 | 52.3 | 60.6 | 23.4 | 20.3 | 26.9 |
|  | 2017 | 30.8 | 28.7 | 32.9 | 66.0 | 62.1 | 69.6 | 65.2 | 61.2 | 68.9 | 34.0 | 30.3 | 37.8 |
| **Females** | 2003 | 31.0 | 28.0 | 34.1 | 73.0 | 68.0 | 77.5 | 54.9 | 49.5 | 60.1 | 19.9 | 15.9 | 24.7 |
|  | 2010 | 31.9 | 29.1 | 34.8 | 74.1 | 69.3 | 78.4 | 68.2 | 62.8 | 73.2 | 32.0 | 27.4 | 37.1 |
|  | 2017 | 30.3 | 27.6 | 33.1 | 73.8 | 69.0 | 78.1 | 73.5 | 68.4 | 78.0 | 39.7 | 34.7 | 45.0 |
| **Males** | 2003 | 37.1 | 33.5 | 40.9 | 45.6 | 39.9 | 51.4 | 24.3 | 19.9 | 29.3 | 6.3 | 3.8 | 10.3 |
|  | 2010 | 32.2 | 28.8 | 35.8 | 57.0 | 50.5 | 63.3 | 44.2 | 38.2 | 50.3 | 14.3 | 10.5 | 19.2 |
|  | 2017 | 31.2 | 28.1 | 34.5 | 58.1 | 52.2 | 63.8 | 56.7 | 50.9 | 62.4 | 28.2 | 23.0 | 33.9 |

ENS: Chilean National Health Survey. JNC 7 guideline. Prevalence (SBP/DBP ≥140/90mmHg or current use of antihypertensive treatment). Awareness (prior diagnosis of high blood pressure), treatment (according to antihypertensive ATC codes) and control (SBP/DBP<140/90mmHg); estimated amongst those classed as hypertensive.

### **Table S2. Trends for hypertension prevalence, awareness, treatment and control. Chile ENS2003-2010-2017**

| **Population** | **Year** | **Prevalence** | | | | **Awareness** | | | | **Treatment** | | | | **Control** | | | |
| --- | --- | --- | --- | --- | --- | --- | --- | --- | --- | --- | --- | --- | --- | --- | --- | --- | --- |
|  |  | **OR** | **95% CI** | | **p-value** | **OR** | **95% CI** | | **p-value** | **OR** | **95% CI** | | **p-value** | **OR** | **95% CI** | | **p-value** |
| **All** | 2010 vs 2003 | 0.75 | 0.62 | 0.90 | **0.002** | 1.25 | 0.98 | 1.60 | 0.072 | 1.91 | 1.50 | 2.43 | **<0.001** | 2.07 | 1.52 | 2.82 | **<0.001** |
|  | 2017 vs 2003 | 0.56 | 0.46 | 0.67 | **<0.001** | 1.21 | 0.95 | 1.53 | 0.130 | 2.51 | 1.96 | 3.21 | **<0.001** | 3.61 | 2.65 | 4.91 | **<0.001** |
|  | 2017 vs 2010 | 0.75 | 0.62 | 0.89 | **0.001** | 0.96 | 0.75 | 1.23 | 0.762 | 1.31 | 1.02 | 1.68 | **0.034** | 1.74 | 1.35 | 2.26 | **<0.001** |
| **Females** | 2010 vs 2003 | 0.90 | 0.69 | 1.16 | 0.413 | 1.03 | 0.73 | 1.44 | 0.877 | 1.70 | 1.24 | 2.34 | **0.001** | 1.97 | 1.37 | 2.83 | **<0.001** |
|  | 2017 vs 2003 | 0.65 | 0.51 | 0.84 | **0.001** | 0.98 | 0.69 | 1.38 | 0.892 | 1.99 | 1.43 | 2.77 | **<0.001** | 2.93 | 2.04 | 4.20 | **<0.001** |
|  | 2017 vs 2010 | 0.73 | 0.57 | 0.92 | **0.009** | 0.95 | 0.68 | 1.33 | 0.768 | 1.17 | 0.84 | 1.64 | 0.362 | 1.48 | 1.08 | 2.04 | **0.015** |
| **Males** | 2010 vs 2003 | 0.63 | 0.48 | 0.83 | **0.001** | 1.45 | 1.02 | 2.06 | **0.040** | 2.17 | 1.49 | 3.16 | **<0.001** | 2.29 | 1.21 | 4.33 | **0.011** |
|  | 2017 vs 2003 | 0.48 | 0.37 | 0.63 | **<0.001** | 1.40 | 1.00 | 1.97 | 0.051 | 3.15 | 2.16 | 4.61 | **<0.001** | 5.06 | 2.73 | 9.38 | **<0.001** |
|  | 2017 vs 2010 | 0.77 | 0.59 | 1.00 | **0.046** | 0.97 | 0.68 | 1.39 | 0.868 | 1.45 | 1.00 | 2.11 | **0.048** | 2.21 | 1.41 | 3.46 | **0.001** |

ENS: Chilean National Health Survey. OR: Odds ratio from age-adjusted logistic regression. JNC 7 guideline. Prevalence (SBP/DBP ≥140/90mmHg or current use of antihypertensive treatment). Awareness (prior diagnose of high blood pressure), treatment (according to antihypertensive ATC codes) and control (SBP/DBP<140/90mmHg); estimated amongst those classed as hypertensive.

### **Table S3. Trends for hypertension prevalence, awareness, treatment and control according to the definition of mean systolic and diastolic blood pressure (BP). Chile ENS2003-2010-2017**

|  |  | **First and second BP (original analyses)** | | | | **Second and third BP 2010-2017, second BP 2003** | | | |
| --- | --- | --- | --- | --- | --- | --- | --- | --- | --- |
| **outcome** | **exposure** | **OR** | **95% CI** | | **p-value** | **OR** | **95% CI** | | **p-value** |
| Prevalence | Age | 1.09 | 1.09 | 1.10 | **<0.001** | 1.09 | 1.09 | 1.10 | **<0.001** |
|  | ENS2010 vs 2003 | 0.75 | 0.62 | 0.90 | **0.002** | 0.69 | 0.57 | 0.83 | **<0.001** |
|  | ENS2017 vs 2003 | 0.56 | 0.46 | 0.67 | **<0.001** | 0.53 | 0.44 | 0.63 | **<0.001** |
|  | ENS2017 vs 2010 | 0.74 | 0.62 | 0.89 | **0.001** | 0.77 | 0.64 | 0.92 | **0.004** |
|  | Female vs Male | 0.73 | 0.63 | 0.85 | **<0.001** | 0.71 | 0.61 | 0.83 | **<0.001** |
| Awareness | Age | 1.02 | 1.01 | 1.02 | **<0.001** | 1.02 | 1.01 | 1.03 | **<0.001** |
|  | ENS2010 vs 2003 | 1.26 | 0.99 | 1.61 | 0.065 | 1.37 | 1.07 | 1.75 | **0.012** |
|  | ENS2017 vs 2003 | 1.20 | 0.94 | 1.53 | 0.141 | 1.27 | 0.99 | 1.63 | 0.059 |
|  | ENS2017 vs 2010 | 0.95 | 0.74 | 1.22 | 0.702 | 0.93 | 0.72 | 1.20 | 0.559 |
|  | Female vs Male | 2.27 | 1.86 | 2.77 | **<0.001** | 2.40 | 1.96 | 2.94 | **<0.001** |
| Treatment | Age | 1.05 | 1.04 | 1.06 | **<0.001** | 1.05 | 1.04 | 1.06 | **<0.001** |
|  | ENS2010 vs 2003 | 1.93 | 1.52 | 2.45 | **<0.001** | 2.10 | 1.65 | 2.68 | **<0.001** |
|  | ENS2017 vs 2003 | 2.49 | 1.95 | 3.20 | **<0.001** | 2.73 | 2.11 | 3.52 | **<0.001** |
|  | ENS2017 vs 2010 | 1.29 | 1.01 | 1.66 | **0.042** | 1.30 | 1.00 | 1.68 | **0.048** |
|  | Female vs Male | 2.53 | 2.07 | 3.09 | **<0.001** | 2.74 | 2.23 | 3.37 | **<0.001** |
| Control | Age | 1.02 | 1.01 | 1.03 | **<0.001** | 1.02 | 1.01 | 1.03 | **<0.001** |
|  | ENS2010 vs 2003 | 3.13 | 2.20 | 4.46 | **<0.001** | 3.39 | 2.39 | 4.81 | **<0.001** |
|  | ENS2017 vs 2003 | 6.85 | 4.69 | 10.02 | **<0.001** | 7.58 | 5.18 | 11.08 | **<0.001** |
|  | ENS2017 vs 2010 | 2.19 | 1.55 | 3.08 | **<0.001** | 2.24 | 1.60 | 3.13 | **<0.001** |
|  | Female vs Male | 3.53 | 2.63 | 4.73 | **<0.001** | 3.41 | 2.55 | 4.55 | **<0.001** |

OR: Odds ratio

95% CI: 95% Confidence Interval

### **Figure S1. Prevalence of hypertension (BP≥140/90 mmHg or use of antihypertensive treatment) using to define treatment self-reported data (SR) or** **Anatomical Therapeutic Chemical (ATC) codes from the medicine inventory. Chile ENS2003-2010-2017**


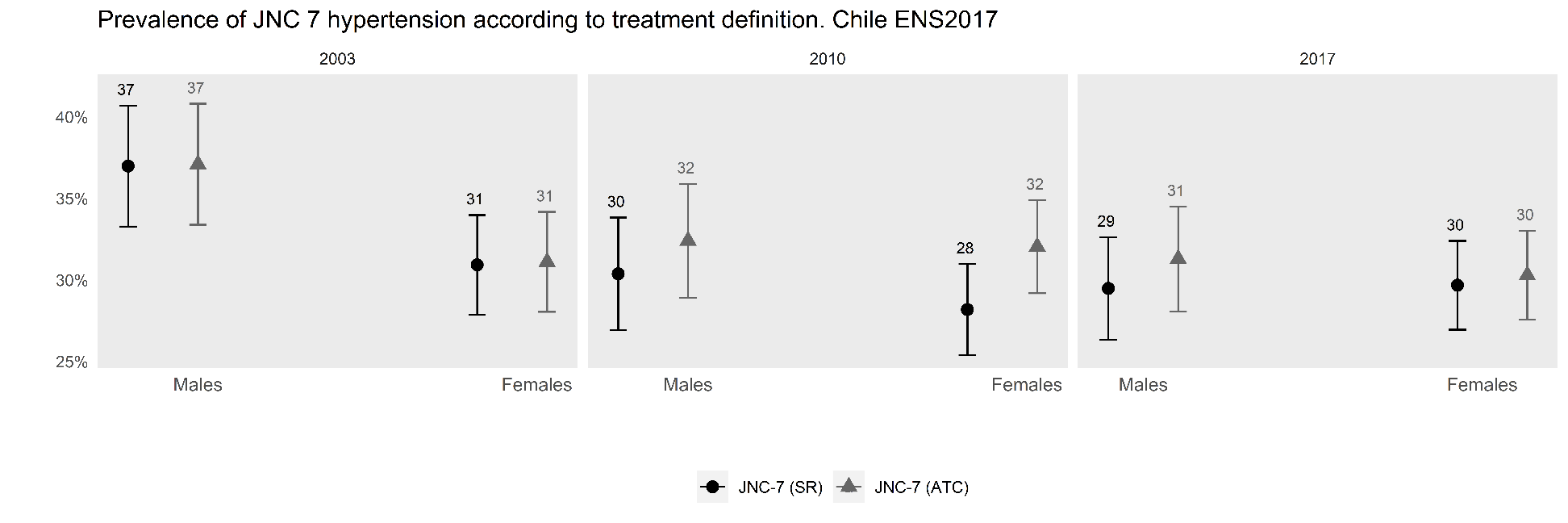


**SR: Self-reported**

**ATC: Anatomical Therapeutic Chemical**

### **Table S4. Blood pressure trends according to treatment status. Chile ENS2003-2010-2017**

| **Treatment** | **Population** | **Trend** | **SBP (mmHg)** | | | | **DBP (mmHg)** | | | |
| --- | --- | --- | --- | --- | --- | --- | --- | --- | --- | --- |
|  |  |  | **β** | **95% CI** | | **p-value** | **β** | **95% CI** | | **p-value** |
| All | Males | ENS2010 vs ENS2003 | -1.07 | -2.70 | 0.56 | 0.200 | -4.15 | -5.29 | -3.02 | **<0.001** |
|  |  | ENS2017 vs ENS2003 | -4.41 | -5.98 | -2.85 | **<0.001** | -6.89 | -8.01 | -5.78 | **<0.001** |
|  |  | ENS2017 vs ENS2010 | -3.35 | -4.92 | -1.77 | **<0.001** | -2.74 | -3.77 | -1.71 | **<0.001** |
|  | Females | ENS2010 vs ENS2003 | -2.66 | -4.21 | -1.11 | **0.001** | -2.91 | -3.96 | -1.86 | **<0.001** |
|  |  | ENS2017 vs ENS2003 | -5.84 | -7.35 | -4.32 | **<0.001** | -4.76 | -5.79 | -3.74 | **<0.001** |
|  |  | ENS2017 vs ENS2010 | -3.17 | -4.64 | -1.71 | **<0.001** | -1.85 | -2.74 | -0.97 | **<0.001** |
| No | Males | ENS2010 vs ENS2003 | -1.70 | -3.32 | -0.09 | **0.039** | -3.99 | -5.16 | -2.81 | **<0.001** |
|  |  | ENS2017 vs ENS2003 | -4.03 | -5.67 | -2.39 | **<0.001** | -6.34 | -7.52 | -5.17 | **<0.001** |
|  |  | ENS2017 vs ENS2010 | -2.32 | -3.92 | -0.73 | **0.004** | -2.36 | -3.46 | -1.25 | **<0.001** |
|  | Females | ENS2010 vs ENS2003 | -2.41 | -4.08 | -0.74 | **0.005** | -2.17 | -3.35 | -0.99 | **<0.001** |
|  |  | ENS2017 vs ENS2003 | -5.53 | -7.12 | -3.95 | **<0.001** | -3.87 | -4.99 | -2.74 | **<0.001** |
|  |  | ENS2017 vs ENS2010 | -3.12 | -4.70 | -1.54 | **<0.001** | -1.69 | -2.71 | -0.68 | **0.001** |
| Yes | Males | ENS2010 vs ENS2003 | -0.55 | -8.93 | 7.83 | 0.898 | -7.74 | -12.90 | -2.58 | **0.003** |
|  |  | ENS2017 vs ENS2003 | -9.76 | -17.38 | -2.13 | **0.012** | -11.21 | -16.21 | -6.20 | **<0.001** |
|  |  | ENS2017 vs ENS2010 | -9.21 | -15.43 | -2.98 | **0.004** | -3.47 | -6.56 | -0.37 | **0.028** |
|  | Females | ENS2010 vs ENS2003 | -4.39 | -8.86 | 0.07 | 0.054 | -5.81 | -9.06 | -2.56 | **<0.001** |
|  |  | ENS2017 vs ENS2003 | -4.33 | -9.61 | 0.95 | 0.108 | -6.56 | -9.73 | -3.40 | **<0.001** |
|  |  | ENS2017 vs ENS2010 | 0.06 | -4.82 | 4.94 | 0.981 | -0.76 | -3.16 | 1.64 | 0.536 |

ENS: Chilean National Health Survey. β: age-adjusted linear regression coefficient. Treatment according to antihypertensive ATC codes. SBP: systolic blood pressure, DBP: Diastolic blood pressure

### **Table S5.** **Prevalence, treatment and control of hypertension according to JNC 7 and 2017 ACC/AHA guidelines. Chile ENS2017**

| **Classification** | **Population** | **Normotensive** | | | | **Untreated uncontrolled** | | | | **Treated uncontrolled** | | | | **Treated controlled** | | | |
| --- | --- | --- | --- | --- | --- | --- | --- | --- | --- | --- | --- | --- | --- | --- | --- | --- | --- |
|  |  | **%** | | **95% CI** | | **%** | | **95% CI** | | **%** | | **95% CI** | | **%** | | **95% CI** | |
| JNC 7 | All | 69.2 | 67.1 | | 71.3 | 10.7 | 9.4 | | 12.2 | 9.6 | 8.5 | | 10.8 | 10.4 | 9.1 | | 11.9 |
|  | Males | 68.8 | 65.5 | | 71.9 | 13.5 | 11.4 | | 16.0 | 8.9 | 7.4 | | 10.8 | 8.8 | 7.0 | | 11.0 |
|  | Females | 69.7 | 66.9 | | 72.4 | 8.0 | 6.5 | | 9.9 | 10.2 | 8.7 | | 12.0 | 12.0 | 10.2 | | 14.1 |
| 2017 ACC/AHA | All | 52.4 | 50.0 | | 54.8 | 27.5 | 25.4 | | 29.8 | 14.0 | 12.6 | | 15.5 | 6.0 | 5.0 | | 7.2 |
|  | Males | 46.5 | 42.8 | | 50.3 | 35.8 | 32.3 | | 39.4 | 13.5 | 11.5 | | 15.9 | 4.2 | 3.0 | | 5.8 |
|  | Females | 58.1 | 55.1 | | 61.0 | 19.7 | 17.4 | | 22.2 | 14.5 | 12.7 | | 16.4 | 7.8 | 6.2 | | 9.7 |

ENS: Chilean National Health Survey. JNC 7 groups: normotensive (<140/90mmHg); treated and controlled (<140/90 mmHg); treated and uncontrolled (≥140/90 mmHg); untreated and uncontrolled (≥140/90 mmHg). 2017 ACC/AHA groups: normotensive (<130/80 mmHg); treated and controlled (<130/80 mmHg); treated and uncontrolled (≥130/80 mmHg); untreated and uncontrolled (≥130/80 mmHg). Treated or untreated according to antihypertensive ATC codes.
